# Influence of Fungicide Application on Rhizosphere Microbiota Structure and Microbial Secreted Enzymes in Diverse Cannabinoid-Rich Hemp Cultivars

**DOI:** 10.3390/ijms25115892

**Published:** 2024-05-28

**Authors:** Junhuan Xu, Tyson Knight, Donchel Boone, Muhammad Saleem, Sheree J. Finley, Nicole Gauthier, Joseph A. Ayariga, Rufus Akinrinlola, Melissa Pulkoski, Kadie Britt, Tigist Tolosa, Yara I. Rosado-Rivera, Ibrahim Iddrisu, Ivy Thweatt, Ting Li, Simon Zebelo, Hannah Burrack, Lindsey Thiessen, Zachariah Hansen, Ernest Bernard, Thomas Kuhar, Michelle Samuel-Foo, Olufemi S. Ajayi

**Affiliations:** 1 The Industrial Hemp Program, Alabama State University, 1627 Harris Way, Montgomery, AL 36104, USAjayariga@alasu.edu (J.A.A.);; 2 Department of Biological Sciences, Alabama State University, 1627 Harris Way, Montgomery, AL 36104, USA; 3 Department of Physical and Forensic Sciences, Alabama State University, 915 S. Jackson Street, Montgomery, AL 36104, USA; 4 Department of Plant Pathology, University of Kentucky, 201F Plant Science Building, Lexington, KY 40546, USA; ngauthier@uky.edu; 5 Department of Entomology and Plant Pathology, University of Tennessee, Knoxville, TN 37996, USA; 6 Department of Crop Science, North Carolina State University, Raleigh, NC 27962, USA; 7 Department of Entomology, Virginia Polytechnic Institute and State University, 170 Drillfield Drive, 220 Price Hall, Blacksburg, VA 24061, USA; kadieb@vt.edu (K.B.);; 8 Department of Agriculture Food and Resource Sciences, University of Maryland Eastern Shore, Princess Anne, MD 21853, USA; 9 Department of Entomology, Michigan State University, East Lansing, MI 48824, USA; 10 USDA-APHIS-PPQ, Raleigh, NC 27606, USA; 11 Emerging Pests and Pathogens Research Unit, USDA-ARS, 538 Tower Rd., Ithaca, NY 14850, USA

**Keywords:** *Cannabis sativa*, cannabinoid (CBD), diversity, abundance, hemp rhizosphere, fungicide, enzymes

## Abstract

Microbes and enzymes play essential roles in soil and plant rhizosphere ecosystem functioning. However, fungicides and plant root secretions may impact the diversity and abundance of microbiota structure and enzymatic activities in the plant rhizosphere. In this study, we analyzed soil samples from the rhizosphere of four cannabinoid-rich hemp (*Cannabis sativa*) cultivars (Otto II, BaOx, Cherry Citrus, and Wife) subjected to three different treatments (natural infection, fungal inoculation, and fungicide treatment). DNA was extracted from the soil samples, 16S rDNA was sequenced, and data were analyzed for diversity and abundance among different fungicide treatments and hemp cultivars. Fungicide treatment significantly impacted the diversity and abundance of the hemp rhizosphere microbiota structure, and it substantially increased the abundance of the phyla Archaea and Rokubacteria. However, the abundances of the phyla Pseudomonadota and Gemmatimonadetes were substantially decreased in treatments with fungicides compared to those without fungicides in the four hemp cultivars. In addition, the diversity and abundance of the rhizosphere microbiota structure were influenced by hemp cultivars. The influence of Cherry Citrus on the diversity and abundance of the hemp rhizosphere microbiota structure was less compared to the other three hemp cultivars (Otto II, BaOx, and Wife). Moreover, fungicide treatment affected enzymatic activities in the hemp rhizosphere. The application of fungicides significantly decreased enzyme abundance in the rhizosphere of all four hemp cultivars. Enzymes such as dehydrogenase, dioxygenase, hydrolase, transferase, oxidase, carboxylase, and peptidase significantly decreased in all the four hemp rhizosphere treated with fungicides compared to those not treated. These enzymes may be involved in the function of metabolizing organic matter and degrading xenobiotics. The ecological significance of these findings lies in the recognition that fungicides impact enzymes, microbiota structure, and the overall ecosystem within the hemp rhizosphere.

## 1. Introduction

Microorganisms play pivotal roles in ecosystem functioning within both soil and plant rhizospheres [1]. They facilitate the transformation of organic matter [2], nutrient release [3], and xenobiotic degradation [4] through biologically and biochemically mediated processes. However, the application of pesticides can disrupt microbial communities and soil ecosystems and reduce microbial diversity and abundance in the plant rhizosphere. This is primarily due to the adverse effects of pesticides on microbial proliferation, biotransformation, nitrogen fixation, phosphorus solubilization, and other vital soil functions [5,6]. For example, Azoxystrobin, a widely used fungicide derived from strobilurin, is commonly employed for controlling fungal plant pathogens [6]. Its broad-spectrum activity makes it a valuable candidate for studying the impact of fungicides on non-target organisms and the surrounding environment. Azoxystrobin exerts systemic activity against pathogens by inhibiting mitochondrial respiration. Specifically, it binds to cytochrome b complexes, thereby blocking electron transport from cytochrome b to c. This interruption in electron transport inhibits the generation of energy through oxidative phosphorylation, leading to pathogen death [7]. While the active substance of Azoxystrobin can be degraded by microorganisms and enzymes, it also negatively impacts soil microbial diversity, abundance, and enzymatic activity [5,6].

Fungal diseases impact many economically important crops including hemp. In the United States, fungal diseases of hemp include root-infecting pathogens such as *Fusarium oxysporum*, *F. solani*, *F. brachygibbosum*, *Pythium dissotocum*, *P. myriotylum*, and *P. aphanidermatum*, along with foliar disease caused by *Golovinomyces cichoracearum* [8]. Unlike most other crops, hemp does not have sufficient labeled fungicides for managing these fungal diseases. While common management strategies in other crops involve applying fungicides during the growing season [9], the limited availability of labeled fungicides poses challenges for disease control in hemp. Moreover, frequent use of fungicides may have adverse effects on the natural environment by impacting the structure of soil-dwelling microbiota and biochemical processes [9,10,11,12,13]. Fungicides that negatively influence microbial diversity and abundance can damage the soil ecosystem, thus posing sustainability concerns for soil use [14,15,16]. Therefore, it is crucial to strike a balance between protecting beneficial microbial communities and controlling diseases when applying fungicides in hemp cultivation.

Enzymatic and metabolic activities within the soil can be affected by fungicides [16]. Enzymes play essential roles in various soil processes and serve as environmental biomarkers in response to soil stress [17]. They catalyze numerous biochemical processes, including element cycling, organic compound mineralization, and pollutant transformation [18]. Fungicides can interact with enzymes such as ureases, dehydrogenases, proteases, phosphatases, or β-glucosidase, leading to their binding with the active centers of these enzymes in the soil and affecting their catalytic activities [16]. Changes in enzyme activities can influence nutrient availability to plants, thereby enhancing soil fertility and improving soil ecosystem health [19]. Despite the recent resurgence in hemp cultivation due to the lifting of bans in many states across the United States [20,21,22,23], there remains a noticeable scarcity of research focusing on the impact of various fungicides on the diversity and abundance of microbiota structure in the hemp rhizosphere and their effects on enzymatic activities [24]. Furthermore, knowledge gaps persist regarding the status of soil microbiota structure influenced by different hemp plant cultivars and fungicide treatments. We hypothesized that fungicides and different hemp cultivars have significant effects on the diversity and abundance of microbiota and enzymes in the hemp rhizosphere. Our objective was to investigate how different fungicide treatments and various hemp cultivars influence microbial diversity, abundance, and enzymatic activities within the hemp rhizospheres.

## 2. Results

### 2.1. Overview of the Sequence Data and Treatments

**Figure 1:**
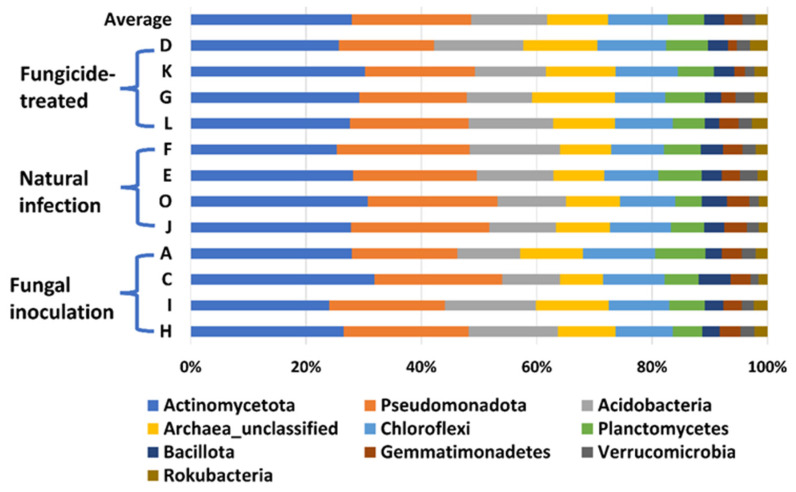
The relative abundance of the top 10 (out of 31) phyla of bacteria on the hemp rhizosphere at different treatments. Note: Otto II, BaOx, Wife, Cherry Citrus with fungicide treatment are denoted by D, K, G, L, respectively. Otto II, BaOx, Wife, Cherry Citrus with natural infection are represented by F, E, O, J, and Otto II, BaOx, Wife, Cherry Citrus with fungal inoculation are indicated by A, C, I, H.

In this study, a total of 3,181,725 counts of sequences, a minimum of 27,493 to a maximum of 75,339 counts per sample with an average of 66,285.9 ± 1501, were obtained. A total of 22,548 feature IDs were identified from 48 samples (Supplementary Table S1). Among them, 21,857 out of 22,548 feature IDs (96.9%) belong to bacterial phyla, 207 (0.9%) belong to archaea-unclassified, and 484 (2.1%) are unclassified (Figure 1). The ten most abundant phyla are Actinomycetota, Pseudomonadota, Acidobacteria, Archaea_unclassified, Chloroflexi, Planctomycetes, Bacillota, Gemmatimonadetes, Verrucomicrobia, and Rokubacteria (Table 1, Figure 1).

### 2.2. Impacts of Fungicides and Hemp Cultivars on Hemp Rhizosphere Microbial Diversity

Alpha diversity metrics, including Chao1, Observed species, Goods_coverage, Shannon, and Simpson, demonstrate high species richness and evenness (Supplementary Table S2). Chao1 estimates total species richness, with a range of 810 to 2955 across samples. Observed species counts the distinct species present, ranging from 807 to 2710. Goods_coverage measures the proportion of Observed species diversity, ranging from 0.97 to 1.00. Shannon calculates species diversity, with values ranging from 8.77 to 10.43. Simpson measures species evenness, with values ranging from 0.99 to 1.00, indicating diverse and evenly distributed species within samples.

**Figure 2:**
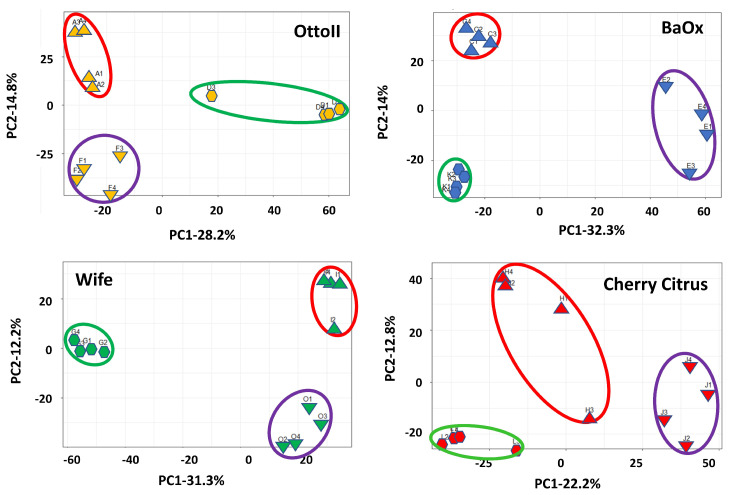
Principal component analysis (PCA) of the relative abundance of the 42 samples across the 3 fungicide treatments and 4 hemp cultivars. Note: Otto II, BaOx, Wife, Cherry Citrus with fungicide treatment are denoted by D, K, G, L (in the green circle), respectively. Otto II, BaOx, Wife, Cherry Citrus with natural infection are represented by F, E, O, J (in the purple circle), and Otto II, BaOx, Wife, Cherry Citrus with fungal inoculation are indicated by A, C, I, H (in the red circle).

Principal component analysis (PCA) showed that two principal components accounted for 43.0%, 46.3%, 35.0%, and 43.5%, with the most variation (28.2%, 32.3%, 22.2, and 31.3%, respectively) explained by PC1 and the second most variation (14.9%, 14.0%, 12.8%, and 12.2% respectively) explained by PC2, showing a separation by different treatments (fungicide-treated, fungal inoculation, and natural infection) with four different cultivars (Otto II, BaOx, Wife, and Cherry Citrus) for the hemp rhizosphere microbial community (Figure 2). The findings indicate that treatments with different replicates displayed similar microbial composition structures, while distinct cultivars treated with fungicide exhibited varying microbial composition structures. The second component exhibited a positive correlation with all samples subjected to fungal inoculations, while displaying a negative correlation with samples affected by natural infections. Furthermore, the correlation patterns between samples from each treatment and hemp cultivar (BaOx, Wife, Cherry Citrus, and Otto2) differed. The PCA results from the data analysis reveal clear patterns linked to fungicide and non-fungicide treatments along the first component axis. Furthermore, factors concerning fungal inoculation and natural infection are evident along the second component axis (Figure 2).

**Figure 3:**
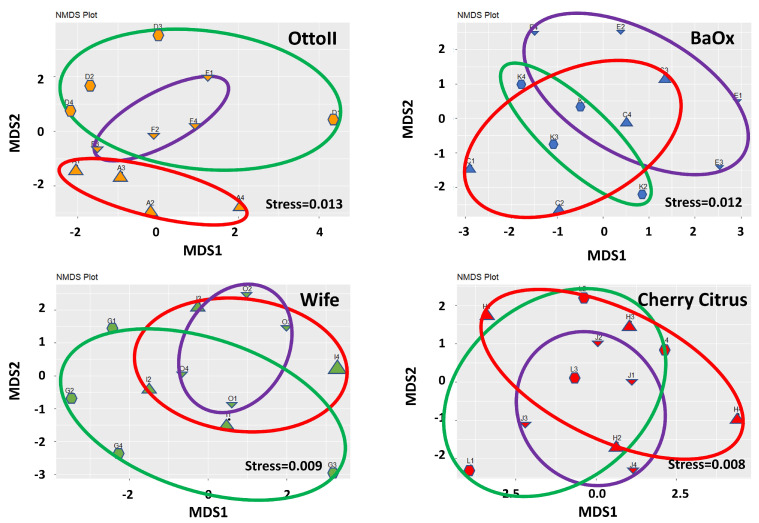
Non-metric multidimensional scaling (NMDS) of the relative abundance of the 42 samples across the 3 fungicide treatments and 4 hemp cultivars. Note: Otto II, BaOx, Wife, Cherry Citrus with fungicide treatment are denoted by D, K, G, L (in the green circle with pentagon symbols), respectively. Otto II, BaOx, Wife, Cherry Citrus with natural infection are represented by F, E, O, J (in the purple circle with downward triangle symbols), and Otto II, BaOx, Wife, Cherry Citrus with fungal inoculation are indicated by A, C, I, H (in the red circle with upward triangle symbols).

The results of non-metric multidimensional scaling analysis (NMDS) demonstrate that the differences between samples within each treatment group are effectively captured and represented in the lower-dimensional space (Figure 3). The stress values of 0.013 for OttoII, 0.012 for BaOx, 0.009 for Wife, and 0.008 for Cherry Citrus indicate that the NMDS representation of the data for each treatment is highly satisfactory.

**Figure 4:**
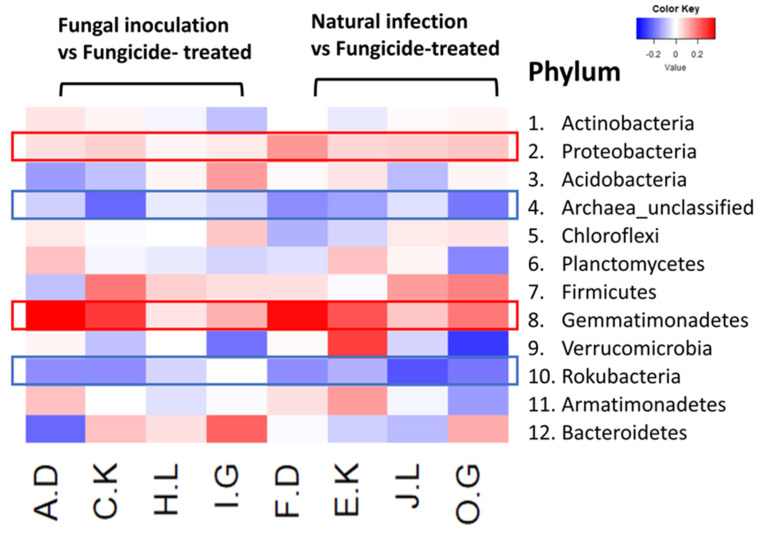
The relative abundance (RA) of the top 12 (out of 31) phyla between fungicide-treated and other treatments in the hemp rhizosphere. Note: The ratios of fungicide-treated to fungal inoculation for the hemp cultivars Otto II, BaOx, Cherry Citrus, Wife are represented by A.D, C.K, H.L, I.G, while the ratios of fungicide-treated to natural infection for the same cultivars are denoted by F.D, E.K, J.L, O.G. Two color scales are utilized, with blue indicating low abundance and red indicating high abundance in fungicide-treated samples compared to fungal inoculation or natural infection.

The heatmap analysis revealed notable changes in the relative abundance of phyla within the hemp rhizosphere. Specifically, the abundance of Rokubacteria significantly increased in hemp cultivars treated with fungicides compared to those subjected to non-fungicide treatments, including fungal inoculation and natural infection control. Conversely, the relative abundance of Pseudomonadota and Gemmatimonadetes markedly decreased in fungicide-treated hemp cultivars compared to non-fungicide-treated counterparts, including those inoculated with fungi and natural infection controls (Figure 4). Furthermore, different hemp cultivars exerted varying effects on the relative abundance of several phyla, including Actinomycetota, Acidobacteria, Chloroflexi, Planctomycetes, Bacillota, Verrucomicrobia, Armatimonadetes, and Bacteroidota (Figure 4).

### 2.3. The Abundance of Microbial Communities in Hemp Rhizosphere across Hemp Cultivars

**Figure 5:**
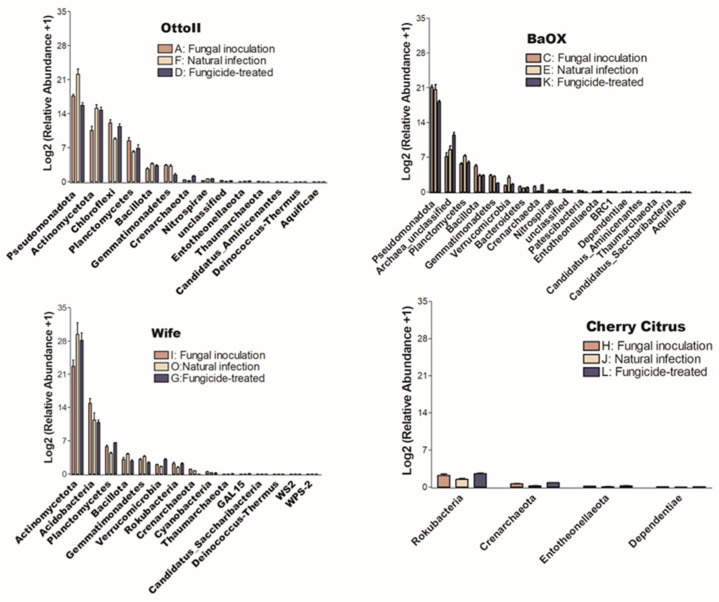
The relative abundance of bacteria that significantly varies among three distinct treatments in the rhizosphere of four hemp cultivars. The treatments include fungal inoculation (A, C, I, H), natural infection (F, E, O, J), and fungicide treatment (D, K, G, L) across the hemp cultivars Otto II, BaOx, Wife, and Cherry Citrus. The vertical axis represents the relative abundance [Log2 (relative abundance +1)] of each bacterial phylum, while the horizontal axis displays all microbial phyla that exhibit significant differences across the three treatments within the rhizosphere (*P* < 0.05). The four hemp cultivars are clearly labeled for easy identification. Overall, this figure illustrates the dynamic microbial composition within hemp plant rhizospheres under varying treatment conditions, emphasizing the specific bacterial phyla that significantly respond to these treatments across the four cultivars.

The number of phyla that showed significant differences (*P* < 0.05) in the abundance of microbial communities among the three treatments in each of the four diverse cannabinoid-rich hemp cultivars is as follows: 14, 18, 15, and 4 in cultivars Otto II, BaOx, Wife, and Cherry Citrus, respectively (Figure 5). Of these phyla, only Crenarchaeota is shared among the four hemp cultivars. The Entotheonellaeota phylum was shared among three hemp cultivars (Otto II, BaOx, and Cherry Citrus). Three phyla (Bacillota, Gemmatimonadetes, and Thaumarchaeota) were shared among three hemp cultivars (Otto II, BaOx, and Wife). Comparing the impact of fungicide treatment on the relative abundance of microbial communities across the four hemp cultivars, Cherry Citrus exhibited the lowest levels. Only four phyla (Crenarchaeota, Dependentiae, Entotheonellaeota, and Rokubacteria) displayed notable differences in microbial community abundance, as depicted in Figure 5.

### 2.4. The Abundance of Microbial Communities in Hemp Rhizosphere of Different Cultivars across Fungicide Treatments

**Figure 6:**
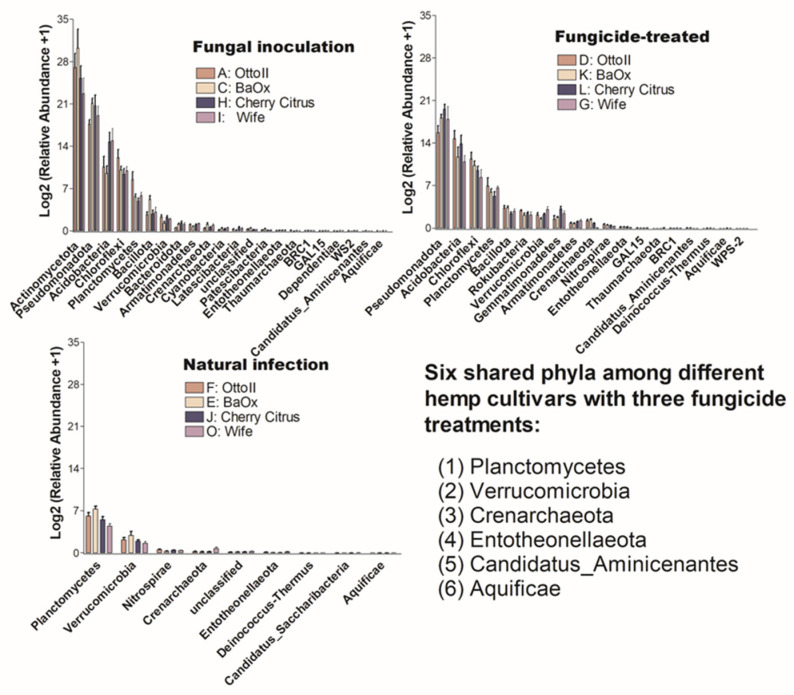
The relative abundance of bacteria that significantly varies across four hemp cultivars in the rhizosphere under three treatments. These cultivars are Otto II, BaOx, Wife, and Cherry Citrus, subjected to fungal inoculation (A, C, H, I), fungicide treatment (D, K, L, G), and natural infection (F, E, J, O). The vertical axis displays the relative abundance [Log2 (relative abundance +1)] of each bacterial phylum, while the horizontal axis lists the microbial phyla with significant differences among the cultivars under the three treatments (*P* < 0.05). The legend clarifies the three treatments (fungal inoculation, fungicide treatment, and natural infection). This figure provides insights into distinct bacterial populations in the rhizosphere of different hemp cultivars under varied treatment conditions, highlighting significant variations in bacterial abundance among cultivars across the treatments.

The number of phyla that showed significant differences (*P* < 0.05) in the abundance of microbial communities among the four diverse cannabinoid-rich hemp cultivars in each of the three treatments is as follows: 19, 22, and 9 phyla in the treatments of fungal inoculation, fungicide treatment, and natural infection, respectively (Figure 6). Comparing the relative abundance of microbial communities among the three fungicide treatments, it was observed that the natural infection treatment had the lowest number of phyla that showed significant differences in relative abundance among different cultivars. Six phyla (Aquificae, Candidatus_Aminicenantes, Crenarchaeota, Entotheonellaeota, Planctomycetes, and Verrucomicrobia) were shared among the three fungicide treatments (Figure 6).

### 2.5. Fungicide Affects Abundance of Clusters of Orthologous Genes in Hemp Rhizosphere

**Figure 7:**
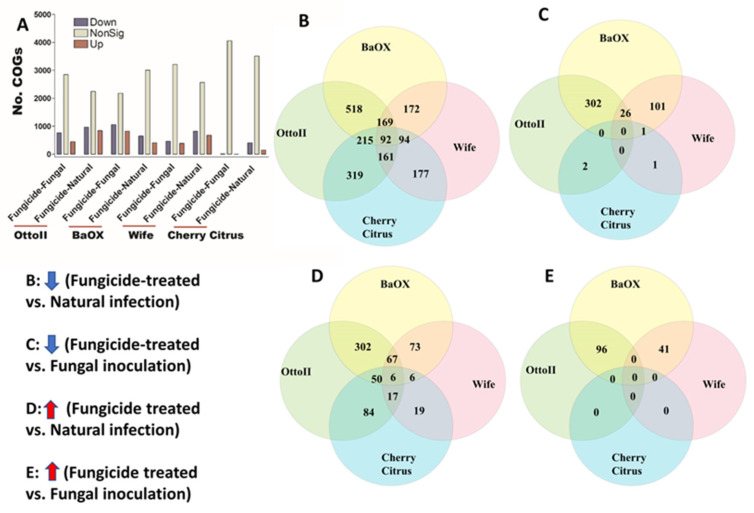
The relative abundance of Clusters of Orthologous Genes (COGs) and a Venn diagram highlighting shared COGs that show significant differences between fungicide-treated and non-fungicide-treated for the four hemp cultivars. (**A**): This panel displays the number of COGs that are significantly down-regulated (down) and up-regulated (up) (*P* < 0.05) or non-significantly regulated (nonSig) (*P* < 0.05) between fungicide-treated versus fungal inoculation, or fungicide-treated versus natural infection, across the four hemp cultivars (e.g., Otto II, BaOx, Wife, and Cherry Citrus). (**B**) shows the shared COGs significantly down-regulated in fungicide treatment compared to natural infection across the four hemp cultivars. (**C**) depicts the shared COGs significantly down-regulated in fungicide treatment compared to fungal inoculation for the four hemp cultivars. (**D**) highlights the shared COGs significantly up-regulated in fungicide treatment compared to natural infection for the four hemp cultivars. (**E**) presents the shared COGs significantly up-regulated in fungicide treatment compared to fungal inoculation for the four hemp cultivars. These insights offer a detailed view of how fungicide treatment influences the regulation of COGs within different hemp cultivars, shedding light on specific genetic responses to fungicide exposure.

We identified 4513 COGs (Clusters of Orthologous Genes) involved in the function of metabolic, energy, signal transduction, transport, defense, transcription, translation, and other pathway genes from the rhizosphere of the four hemp cultivars with three fungicide treatments. Across the four hemp cultivars and comparing the fungicide treatment to one of the two non-fungicide treatments (i.e., fungal inoculation and natural infection), at least 3396 out of 4513 COGs were significantly different (i.e., both higher and lower) in abundance (FDR < 0.05) in the hemp rhizospheres (Supplementary Table S3). When comparing the abundance of COGs between the fungicide treatment and a non-fungicide treatment for each of the four hemp cultivars, there was a greater number of genes that exhibited a significant decrease compared to those that showed a significant increase (Figure 7). Comparing COG abundance between the fungicide treatment and the natural infection treatment for each of the four hemp cultivars, there were more COGs found to be significantly decreased (92 genes) than those that were significantly increased (6 genes) (Figure 7B,D). Comparing COG changes among the four hemp cultivars, Cherry Citrus had the least number (FDR < 0.05) (Figure 7A). There was no shared COG found between the fungicide treatment and the fungal-inoculation treatment among the four hemp cultivars (Figure 7C,E). The description of some of the decreased shared COGs include the Phage terminase large subunit, Predicted periplasmic protein, Capsule polysaccharide export protein KpsE/RkpR, Carbonic anhydrase, Inner membrane protein involved in colicin E2 resistance, Succinylarginine dihydrolase, Periplasmic regulator RcnB of Ni and Co efflux, Murein tripeptide amidase MpaA, Predicted aspartyl protease, Protein required for attachment to host cells, Cytochrome c556, and several Mu-like prophage proteins and Uncharacterized proteins (Table 2).

### 2.6. Fungicide Affects Abundance of Enzymes in Hemp Rhizosphere

**Figure 8:**
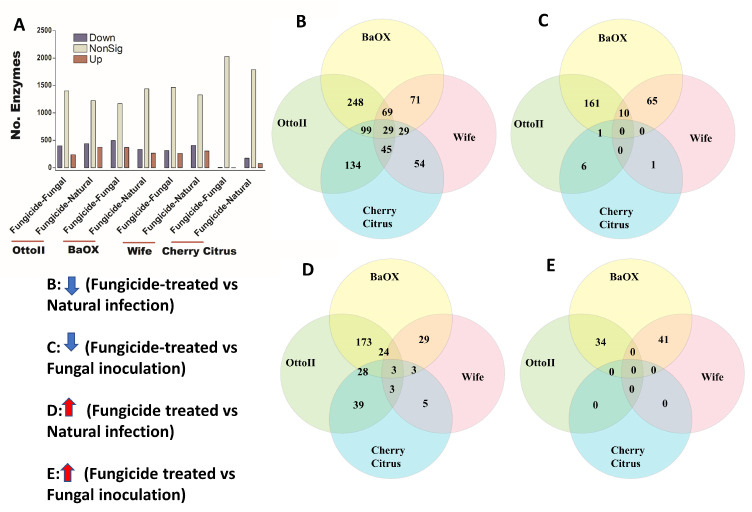
The relative abundance of enzymes and a Venn diagram illustrating shared enzymes that exhibit significant differences between fungicide-treated and non-fungicide-treated hemp cultivars. (**A**): This panel quantifies the number of enzymes that are significantly down-regulated (down) and up-regulated (up) (*P* < 0.05) or non-significantly regulated (nonSig) (*P* < 0.05) between fungicide-treated versus fungal inoculation, or fungicide-treated versus natural infection, across the four hemp cultivars (e.g., Otto II, BaOx, Wife, and Cherry Citrus). (**B**) demonstrates the shared enzymes significantly down-regulated in fungicide treatment compared to natural infection across the four hemp cultivars. (**C**) illustrates the shared enzymes significantly down-regulated in fungicide treatment versus fungal inoculation for the four hemp cultivars. (**D**) highlights the shared enzymes significantly up-regulated in fungicide treatment compared to natural infection for the four hemp cultivars. (**E**) presents the shared enzymes significantly up-regulated in fungicide treatment compared to fungal inoculation for the four hemp cultivars. These insights offer a detailed look at how fungicide treatment affects enzyme regulation in diverse cannabinoid-rich hemp cultivars, revealing specific enzyme responses to fungicide exposure.

We identified 2439 enzymes including transferases, hydrolases, lyases, isomerases, ligases, and translocases from the rhizosphere of the four hemp cultivars with the three different treatments. Across the four hemp cultivars and comparing the fungicide treatment to one of the two non-fungicide treatments (i.e., fungal inoculation and natural infection), statistical analysis revealed that at least 1709 out of 2439 enzymes were significantly different (i.e., both higher and lower) in abundance in the hemp rhizospheres estimated by R statistical (FDR < 0.05) (Supplementary Table S4). When comparing the abundance of enzymes between the fungicide treatment and a non-fungicide treatment for each of the four hemp cultivars, a higher number of enzymes were observed to significantly decrease than increase (Figure 8; Supplementary Table S4). Comparing enzyme changes among the four hemp cultivars, Cherry Citrus had the least number (with FDR < 0.05) (Figure 8A). When comparing enzyme abundance between the fungicide treatment and the natural infection treatment for each of the four hemp cultivars, it was found that 29 enzymes significantly decreased in the fungicide treatment, while 3 enzymes increased in the fungicide treatment compared to the natural infection treatment (Figure 8B,D). There was no shared enzyme found between the fungicide treatment and non-fungicide treatment control (the fungal-inoculation treatment) among the four hemp cultivars (Figure 8C,E). Some of these decreased shared enzymes are RNA ligase (ATP), geranylgeranyl diphosphate synthase, Phosphoglycerate mutase (2,3-diphosphoglycerate-independent), Serine 3-dehydrogenase (NADP(^+^)), Succinylglutamate-semialdehyde dehydrogenase, Succinate-semialdehyde dehydrogenase (NAD(^+^)), N-succinylarginine dihydrolase, D-arginine dehydrogenase, Arabinan endo-1,5-alpha-L-arabinosidase, Bilirubin oxidase, and some other dehydrogenases, transferases, peptidases, synthases, dioxygenase, isomerase, racemase, transaminase, hydrolases, carboxylase, oxidase, amidase, and exodeoxyribonuclease (Table 3).

### 2.7. Fungicide Affects Abundance of Pathways in Hemp Rhizosphere

**Figure 9:**
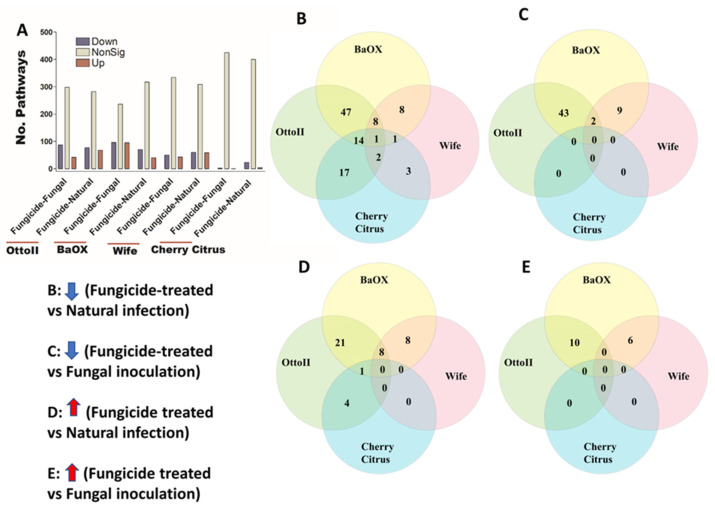
The relative abundance of pathways and a Venn diagram depicting shared pathways that display significant differences between fungicide-treated and non-fungicide-treated hemp cultivars. (**A**): This panel quantifies the number of pathways significantly down-regulated (down) and up-regulated (up) (*P* < 0.05) or non-significantly regulated (nonSig) (*P* < 0.05) between fungicide-treated versus fungal inoculation, or fungicide-treated versus natural infection, across the four hemp cultivars (e.g., Otto II, BaOx, Wife, and Cherry Citrus). (**B**) demonstrates the shared pathways significantly down-regulated in fungicide treatment compared to natural infection across the four hemp cultivars. (**C**) illustrates the shared pathways significantly down-regulated in fungicide treatment compared to fungal inoculation for the four hemp cultivars. (**D**) highlights the shared pathways significantly up-regulated in fungicide treatment compared to natural infection for the four hemp cultivars. (**E**) presents the shared pathways significantly up-regulated in fungicide treatment compared to fungal inoculation for the four hemp cultivars. These insights provide a thorough understanding of how fungicide treatment affects the regulation of pathways in various hemp cultivars, offering specific insights into how pathways respond to fungicide exposure. The relative abundance of pathways and the Venn diagram of shared pathways significantly differed between fungicide-treated and non-fungicide-treated in the different hemp cultivars.

We identified 448 pathways involving the metabolism of different chemical compounds in the rhizosphere of the four hemp cultivars with the three different treatments. Across the four hemp cultivars and comparing the fungicide treatment to one of the two non-fungicide treatments (i.e., fungal inoculation and natural infection), statistical analysis showed that the pathway abundance of 329 out of 448 pathways was significantly different in the hemp rhizospheres (FDR < 0.05) (Supplementary Table S5). When comparing the abundance of pathways between the fungicide treatment and a non-fungicide treatment for each of the four hemp cultivars, a higher number of pathways were identified as significantly decreased compared to those that showed a significant increase (FDR < 0.05) (Figure 9A; Supplementary Table S5). Cherry Citrus had the least number of changes in pathways (FDR < 0.05) (Figure 9A). When comparing the pathway abundance between the fungicide treatment and the natural infection treatment for each of the four hemp cultivars, a singular pathway exhibited a significant decrease. Conversely, no shared pathway demonstrated a significant increase (Figure 9B,D). There was no shared pathway found between the fungicide treatment and the fungal-inoculation treatment among the four hemp cultivars (Figure 9C,E). The decreased shared pathway is L-tyrosine degradation I, which was significantly decreased in abundance in the hemp rhizosphere for fungicide treatment compared to natural infection among the four hemp cultivars (FDR < 0.05) (Table 4).

## 3. Discussion

Our study reveals that the application of fungicides leads to a reduction in hemp rhizosphere microbial diversity, and alters microbial abundance and community structure [25]. Heatmap analysis indicated a decrease in the relative abundance of the phyla Pseudomonadota and Gemmatimonadetes in hemp cultivars treated with fungicides compared to those subjected to non-fungicide treatments (fungal inoculation and natural infection). This suggests that fungicide use may negatively impact the relative abundance of Pseudomonadota and Gemmatimonadetes. However, fungicide application could have contrasting effects on certain microbial phyla. Specifically, we observed a notable increase in the relative abundance of the phyla Archaea_unclassified and Rokubacteria in fungicide-treated hemp rhizosphere soil samples compared to non-fungicide treatments. This suggests that fungicide application may enhance the relative abundance of Archaea_unclassified and Rokubacteria.

Previous studies reported that fungicide application can suppress soil bacteria such as Pseudomonadota and Gemmatimonadetes [24,26]. Specifically, dimethyl disulfide, a type of fungicide, was found to significantly inhibit the abundance of Pseudomonadota [24]. Pseudomonadota plays a crucial role in soil ecosystems, with many of its members involved in nutrient cycling, including nitrogen fixation, which contributes to essential nutrient availability for plant growth (refer to Supplementary Table S1). However, it is noteworthy that some members of Pseudomonadota can also act as plant pathogens such as *Agrobacterium tumefaciens*, *Ralstonia solanacearum*, *Xanthomonas* spp., and *Erwinia* spp., although our investigation did not detect significant variations or the presence of notable plant pathogens within this phylum (refer to Supplementary Table S1) [24,27]. Ma et al. (2021) observed a significant decrease in the abundance of Pseudomonadota from 25.7% in the non-fungicide treatment control to 0.09% in the fungicide-treated treatment [24]. Similarly, fungicide application has been found to inhibit the phylum of Gemmatimonadetes, which play various roles in plant health, including nutrient cycling, soil structure improvement, disease suppression, and biological nitrogen fixation (refer to Supplementary Table S1 for a list of different species identified within this phylum) [26,28]. Liao et al. (2022) reported that the application of three fungicides (fluazinam, metalaxyl-mancozeb, and carbendazim) significantly decreased Gemmatimonadetes abundance at 6 weeks compared to 3 weeks. In our study, we observed that the application of Azoxystrobin inhibited the relative abundance of both the Pseudomonadota and Gemmatimonadetes phyla.

The application of fungicides can also stimulate certain microbiota phyla, such as Rokubacteria. For instance, seed coat protectants, containing fungicides like Azoxystrobin, fludioxonil, metalaxyl, sedaxane, and thiabendazole, along with insecticides like Imidacloprid and Thiamethoxam, have been widely utilized in agriculture to mitigate biotic and abiotic stresses, while enhancing crop growth, yield, and health [29,30]. Studies have shown that the application of these seed coat protectant fungicides can promote the abundance of Rokubacteria in soil ecosystems [29,30]. Rokubacteria are found across various terrestrial ecosystems globally, including soils, the rhizosphere, volcanic mud, oil wells, aquifers, and the deep subsurface. They play crucial roles in nutrient cycling, organic matter decomposition, and overall ecosystem health maintenance (refer to Supplementary Table S1 for a list of different species identified within this phylum) [31]. Moreover, distinct pesticides or identical pesticides at different concentrations can elicit diverse effects on microbial communities within the same phyla in soil [14,32,33]. For example, the fungicide mancozeb was reported to have a more detrimental effect on actinomycetes populations at higher dose levels than at lower dose levels [34]. Conversely, the abundance of actinomycetes increased with increasing doses of herbicides such as glyphosate, pethoxamid, and terbuthylazine [35,36]. Actinomycetes play various crucial roles in different ecosystems, including the decomposition of organic matter, soil structure improvement, nitrogen fixation, biocontrol, symbiotic relationships, bioremediation, the production of bioactive compounds, and contribution to marine ecosystems [37]. Although herbicides are not intended to be nutrients for bacteria, they can indirectly affect soil bacteria by altering nutrient availability, organic matter content, and other soil factors [37]. In our study, we observed that Azoxystrobin promoted the relative abundance of Archaea_unclassified and Rokubacteria. Hence, while pesticides may directly affect microorganisms with their toxic properties, they can also indirectly impact microbiota by potentially serving as suitable nutrients or energy sources for other microorganisms [38,39].

Plants exert an influence on indigenous soil microbial communities through the secretion of unique compounds via their roots [40]. The composition of root exudates varies not only between plant species but also among different cultivars of the same plant, thereby mediating below-ground interactions among microorganisms and impacting the relative abundance, diversity, and enzymatic activities of microbial communities in the rhizosphere [41]. Plants offer specialized nutrients for microorganisms and secrete unique antimicrobial metabolites in their exudates, such as those found in chamomile, thyme, and eucalyptus. Moreover, crops have been observed to produce components associated with specific microbial taxon abundance [42]. For example, Ahmed et al. (2021) found that certain bacterial and fungal taxa, such as Planctomycetes and Ascomycota, were prevalent and shared across multiple locations where the clonal hemp cultivar TJ’s CBD was grown, indicating a strong association between these taxa and hemp cultivation regardless of location [42]. Our findings suggest that compared to the other three cultivars (Otto II, BaOx, and Wife), the hemp cultivar Cherry Citrus generally demonstrates lesser variations in microbial abundance and enzymatic activity across different treatments within the hemp rhizosphere (Figure 5). Hence, the varying chemical compositions of hemp cultivars, particularly their cannabidiol (CBD) content, may potentially influence microbial diversity and abundance in the hemp rhizosphere [43]. For instance, Cherry Citrus exhibited a lower CBD content (approximately 10–12%) compared to other cultivars BaOx with 12–16% CBD and Otto II with 15.24% CBD [43]. Apart from CBD content, differences in genotype among cultivars, including variations in secondary metabolites, root exudates, and overall plant physiology, can also significantly influence the composition of the rhizosphere microbiome [44]. However, it is important to note that in this study, CBD content and other factors were not specifically assessed for their correlation with soil microbial content.

Enzymes play pivotal roles in numerous soil processes, facilitating organic matter transformation, nutrient release, and the decomposition of chemical compounds, thereby contributing significantly to soil ecosystem functioning [14,15,16]. However, fungicides can potentially disrupt enzyme activity [15,16]. In our study, we observed a reduction in the abundance of several enzymes within the hemp rhizosphere following fungicide treatment compared to the non-fungicide control. The affected enzymes include RNA ligase (ATP), geranylgeranyl diphosphate synthase, phosphoglycerate mutase (2,3-diphosphoglycerate-independent), serine 3-dehydrogenase (NADP(+)), succinylglutamate-semialdehyde dehydrogenase, succinate-semialdehyde dehydrogenase (NAD(+)), N-succinylarginine dihydrolase, D-arginine dehydrogenase, arabinan endo-1,5-alpha-L-arabinosidase, and bilirubin oxidase. Additionally, a significant decrease was observed in various other enzymes, including dehydrogenases, transferases, peptidases, synthases, dioxygenase, isomerase, racemase, transaminase, hydrolases, carboxylase, oxidase, amidase, and exodeoxyribonuclease, within the hemp rhizosphere subjected to fungicide treatment compared to the naturally infected (non-fungicide-treated) rhizosphere.

The decline in these crucial enzymes may profoundly affect hemp growth in the soil, potentially impeding plant development through alterations in soil metabolism and fungicide degradation within the environment. For instance, RNA ligase (ATP), which is potentially released by bacteria, plays a vital role in repairing, splicing, and editing pathways essential for maintaining genomic integrity in soil bacteria [45]. Similarly, the absence of geranylgeranyl diphosphate synthase could lead to reduced leaf steroid content in plants like *Nicotiana attenuata* [46], impacting their physiological processes. Additionally, enzymes like phosphoglycerate mutase, serine 3-dehydrogenase, succinylglutamate-semialdehyde dehydrogenase, succinate-semialdehyde dehydrogenase, and D-arginine dehydrogenase, belonging to the oxidoreductase family, are crucial for various soil ecosystem functions such as organic matter transformation, nutrient cycling, detoxification, and energy production [47,48,49,50,51]. Furthermore, enzymes like arabinan endo-1,5-alpha-L-arabinosidase and bilirubin oxidase contribute to pectin degradation and bilirubin oxidation, respectively, impacting soil ecosystem processes and fungal biology [52,53]. Incorporating gene-level and enzyme data into our discussion enhances our understanding of how fungicide treatment affects microbial ecology, highlighting the potential repercussions of decreased enzyme activity on plant growth, soil health, and overall ecosystem functioning.

## 4. Materials and Methods

### 4.1. Treatments and Soil Sampling Collection

**Figure 10:**
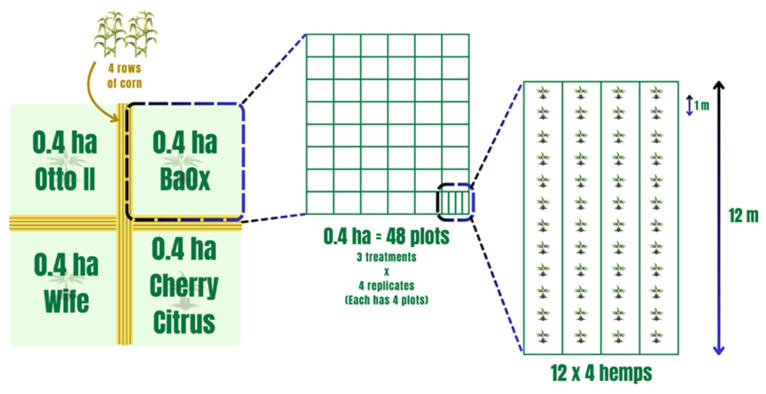
The experimental layout of four hemp cultivars within the field with each subjected to different treatments. Note that the four distinct hemp cultivars—Otto II, BaOx, Wife, and Cherry Citrus—are allocated to individual plots. Within each plot, three distinct treatments are applied to the respective hemp cultivars: fungicide treatment, fungal inoculation, and natural infection. This systematic and methodical design employs a randomized layout to mitigate biases and enhance the reliability of statistical analyses.

Field experiments were conducted at the UK Robinson Center for Appalachian Resource Sustainability in Quicksand, Kentucky (APP), nestled within a horseshoe bend of the north fork of the Kentucky River. The soil in this locale is classified as a Chagrin-Grigsby Complex, characterized by slopes ranging from 0 to 6%, facilitating effective drainage despite intermittent flooding occurrences. This environment, potentially rich in microbial communities, presents an intriguing research setting. The planting design, treatments, and soil sampling collection were as those in Munir et al. 2024 and are as follows [54]. Four hemp cultivars (Otto II, BaOx, Cherry Citrus, and Wife) were selected for this experiment. They are commonly planted hemp varieties for CBD in most parts of the southeastern US, including Kentucky. These cultivars are popular for their high CBD and low Delta-9-tetrahydrocannabinol (also known as THC) content. The four hemp cultivars were arranged in 0.4 ha plots in a split-plot design and replicated four times. Each 0.4 ha field included 48 plots, and each plot contained 48 transplants in four 12 m long rows (refer to Figure 10). The four cultivars were planted on 16 June 2020. Fungicide was applied on 24 July 2020, followed by application of pathogenic fungi on 26 July 2020. Natural infection served as the control. Each of the twelve combination treatments was replicated four times within the field. Four-row corn buffers were planted between blocks to minimize the impact of treatment variables between blocks (refer to Figure 10). For inoculation, the hemp leaves were sprayed with *Bipolaris gigantea*, *Septoria cannabis*, and *Cercospora flagellaris*. These fungi are all leaf spot pathogens. Each inoculation treatment consisted of a combination of three isolates of each disease agent: *Bipolaris gigantea* (isolates 17MA004 and 15JK003), *Cercospora flagellaris* (isolates 19FR003 and 16Hemp001), and *Septoria cannabis* (isolates 18MF001 and 18CL004). All isolates originated from diseased hemp plants from Kentucky and were maintained in 15% glycerol at −80 °C until ready for use. Individual isolates were grown on Potato Dextrose Agar (PDA) spread plates at 23 °C under 12 h fluorescent + black light and 12 h darkness for 7 days. Inoculum suspensions were prepared in the field immediately before inoculation by flooding plates with water and 0.5% Tween 20^®^, dislodging spores with a plastic spatula, and then diluting suspensions to a predetermined concentration. The final concentration was determined post-application: *B. gigantea* 1 × 10^3^, *C. flagellaris* 2 × 10^4^, *S. cannabis* 2 × 10^5^. Inoculum suspensions were applied with a handheld pump sprayer. A total of 6 L of spore suspension was applied to each plot for complete canopy coverage but without runoff. Inoculations occurred during the evenings after temperatures dropped below 25 °C and there was no rain forecasted for at least 24 h [54].

Fungicide treatments commenced approximately 4 weeks following planting. The fungicide Quadris Flowable^®^ (azoxystrobin, Syngenta, Wilmington, DE, USA) was administered at APP, with a dosage of 6 oz per acre (equivalent to 219 mL per hectare) dissolved in 30 gallons per acre of water (approximately 280 L per hectare). Fungicide spraying took place on days when no rain was forecasted for at least 24 h. Natural infection, without the application of spray fungi or fungicides, served as the positive control.

Soils were randomly collected from four plants of each cultivar at APP when the plants were in full flower (29 July 2020). For each collection, the soil was collected from the immediate vicinity of the hemp roots to a depth of 10 cm within 10 cm in diameter of the selected plant. This is because a 10 cm depth within a 10 cm diameter in the hemp is regarded as the zone of soil surrounding a plant root where the biology and chemistry of the soil are influenced by the root and is the dynamic interface among plant roots, soil microbes, fauna, and the soil itself. The collected soil is placed in a Ziploc bag, kept with ice, and shipped overnight from the University of Kentucky to the Industrial Hemp Program lab at Alabama State University. In all, a total of forty-eight soil samples (3 treatments × 4 hemp cultivars × 4 replicates) were collected and stored at −20 °C before DNA extraction.

### 4.2. DNA Extraction

DNA was extracted from our soil samples with the Macherey-Nagel NucleoSpin^®^ Soil DNA Isolation Kit (catalog number NC0706445; Fisher Scientific Company, LLC., Pittsburgh, PA, USA), using the manufacturer’s instructions with the following modifications. A total of 0.5 g of rhizosphere soil was used per soil DNA extraction. Lysis buffer SL1 was added to the samples following the recommended protocol. No enhancer was added for lysis condition adjustment (i.e., the SL1 buffer addition). At the stage of eluting DNA, 40 µL buffer SE was used. The purity and concentration of the DNA were measured using NanoDrop One (Thermos Fisher Scientific, Waltham, MA, USA), with a ratio of A260/A280 between 1.8 and 1.9. The A260/A230 ratios were also determined greater than 2.0.

### 4.3. Library Preparation, Primer Design, and Sequencing

The DNA library was prepared via the template out of genomic DNA using region of interest-specific primers. The primers (341F: 5’-CCTACGGGNGGCWGCAG-3’/805R:5’-GACTACHVGGGTATCTAATCC-3’) were designed to target the V3 and V4 regions of 16S rDNA [55]. The PCR reaction solution was composed of 50 ng microbial DNA (5 ng/µL), 5 µL each forward and reverse primer (1 µM), and 12.5 µL 2x Phusion Hot start flex Master Mix, with ddH_2_O added up to 25µL. Thermocycling conditions were 98 °C for 30 s, 35 cycles of 98 °C for 10 s, 54 °C for 30 s, 72 °C for 45 s, and 72 °C for 10 min. PCR products were isolated by 2% agarose gel, followed by purification with AMPure XT beads to remove primers and primer dimer species. (Beckman Coulter Genomics, Danvers, MA, USA). The products were finally quantified by Qubit assay (Invitrogen, USA).

After PCR, the products were analyzed by Agilent 2100 Bioanalyzer (Agilent, USA) and KAPA Library Quantification Kits (Kapa Biosciences, Woburn, MA, USA). Qualified libraries had concentrations > 2 nM. Sequencing was performed with NovaSeq 6000, 2x250bp (NovaSeq 6000 SP Reagent Kit, 500 cycles) platformed at LC Sciences (2575 West Bellfort Street Suite 270, Houston, TX 77054, USA).

### 4.4. Bioinformatic Analyses of the Sequence Data

Once NGS sequencing is completed, raw data are obtained. Subsequently, the paired-end reads are merged with overlap and undergo quality control and Chimera filtering, resulting in high-quality clean data at LC Sciences. Initially, paired-end reads are assigned to respective samples based on their unique barcode and truncated by removing the barcode and primer sequence. FLASH (Fast Length Adjustment of Short reads) is then employed to merge the paired-end reads. Quality filtering of the raw reads is conducted using specific filtering criteria to yield high-quality clean tags, facilitated by fqtrim (FastQ Trimmer) (v0.94). Chimeric sequences are filtered out using Vsearch software (v2.3.4). Subsequently, dereplication is performed using DADA2 (Divisive Amplicon Denoising Algorithm) [56], resulting in the generation of feature tables and feature sequences (see Supplementary Table S1). The core denoising method of DADA2 aims to reconstruct the exact amplicon sequence variants (ASVs) present in a sample from the noisy amplicon sequencing reads. This process removes background noise, significantly enhancing data accuracy, species resolution, and result reliability. Additional steps involve diversity analysis, species classification annotation, and differential analysis.

### 4.5. Taxonomic Classification

We utilized SILVA (release 138, https://www.arb-silva.de/documentation/release-138/) (accessed on 28 September, 2021) and the NT-16S database for taxonomic classification, employing a parameter threshold of confidence level > 0.7. Feature annotation provided abundance data at multiple taxonomic levels, spanning Domain, Phylum, Class, Order, Family, Genus, and Species. Subsequently, these data underwent differential analysis. Our primary focus and research objectives center on the phylum level, with occasional reference to genus-level information. This strategy enables us to target phyla showing significant abundance discrepancies across different treatments and cultivars. In cases where a phylum encompasses both beneficial microbes and pathogenic pathogens, we further investigate genus-level abundance variations across treatments and cultivars.

### 4.6. Diversity and Abundance Analysis of Hemp Rhizosphere Microbial Communities

Alpha diversity and beta diversity were calculated by normalizing to the same sequences randomly. Then according to the SILVA (release 132) classifier, feature abundance was normalized using the relative abundance of each sample. Alpha diversity is applied in analyzing the complexity of species diversity for a sample through 5 indices, including Chao1, Observed species, Goods_coverage, Shannon, and Simpson, which were calculated with QIIME2 (Quantitative Insights Into Microbial Ecology 2). Beta diversity PCA (principal component analysis) and NMDS (non-metric multidimensional scaling) were carried out using the relative abundance of feature sequences between 4 biological replicates of 3 treatments (natural infection, fungal inoculation, and fungicide treatment) and 4 hemp cultivars (Otto II, BaOx, Cherry Citrus, and Wife) by R statistical software (version 4.0.2) vegan and pca3d with ggplot2 [57]. Heatmaps were created comparing the relative abundance of each phylum for the non-fungicide treatment (fungal inoculation and natural infection) minus the relative abundance for the fungicide treatment divided by the relative abundance for fungicide treatment and then analyzed using R package (version 4.0.2) with RColorBrewer, Pheatmap, and ggplot2 [56].

### 4.7. The Analyses of COG and Enzyme Relative Abundance

The limitation of microbial community marker-gene sequencing is its inability to directly reveal the functional composition of sampled communities. PICRUSt (Phylogenetic Investigation of Communities by Reconstruction of Unobserved States) was developed in 2013 to predict the functional potential of microbial communities based on marker gene sequencing profiles [58]. We utilized the recently released PICRUSt2 Version 1 software (https://github.com/picrust/picrust2) to predict functional abundances based on marker gene sequences [59,60]. These functions typically correspond to gene families, such as KEGG orthologs and enzyme classification numbers, although arbitrary properties can also be predicted. From the PICRUSt2 function prediction results, we obtained gene family annotations, including database functions from COG (Clusters of Orthologous Groups), EC (Enzyme Commission numbers), and pathways. These analyses were conducted at L.C. Science, enhancing our understanding of microbial community functions.

### 4.8. Statistical Analysis

The comparison of the relative abundance for COG (Supplementary Table S3), enzymes (Supplementary Table S4), and pathways (Supplementary Table S5) was conducted between fungicide and non-fungicide treatments across 48 samples. These samples comprised 4 replicates each from 3 treatments and 4 diverse cannabinoid-rich hemp cultivars. The analysis was performed using R statistical software version 4.0.2 (R Core Team; https://R-project.org) along with the DESeq2 package version 1.28.1 [61]. Adjusted *P* values (adj. *P*) were determined using the Benjamini–Hochberg method, where adj. *P* < 0.05 indicated significant differences in relative abundance.

The variance in the relative abundance of phyla across various cannabinoid-rich hemp cultivars was assessed through statistical tests. The Kruskal–Wallis test was employed for comparing multiple groups, while the Wilcoxon test was used for paired group comparisons. These analyses were conducted using R statistical software version 4.0.2 with the ggpubr package. Statistically significant results were identified with *P* values below 0.05. Graphical representations of the data were generated using GraphPad Prism Software (GraphPad Software, San Diego, CA, USA).

### 4.9. Data Availability

The data analyzed from this study are available at NCBI (https://submit.ncbi.nlm.nih.gov/subs/genbank/SUB13018760/overview. GenBank submission: SUB13018760 with accession number KHUW00000000).

## 5. Conclusions

Hemp has gained prominence not only for its industrial and medicinal uses but also for its potential to mitigate environmental issues such as soil remediation and carbon sequestration [42,62]. Understanding how fungicides impact the microbial communities associated with hemp is crucial, as these microorganisms play pivotal roles in nutrient cycling, disease resistance, and overall plant health [15,16]. This study underscores the substantial impact of fungicides and hemp variety on the diversity and abundance of the hemp rhizosphere microbial community, shedding light on a critical aspect of hemp cultivation. We observed shifts in the microbial composition, with increased relative abundances of Archaea and Rokubacteria but decreased relative abundances of Pseudomonadota and Gemmatimonadetes. Furthermore, fungicides also influence the enzymatic activity in the hemp rhizosphere, suggesting potential impacts on nutrient cycling and overall soil health. The study also found cultivar-specific variations in microbial communities, particularly in Cherry Citrus, indicating the importance of tailoring cultivation practices to different hemp varieties. This research increases our understanding of hemp rhizosphere microbial dynamics and directs us toward the goal of optimizing hemp cultivation methods and ensuring the sustainability of hemp ecosystems by preserving the delicate balance of microorganisms within the plant’s rhizosphere.

## Figures and Tables

**Table 1:** The relative abundance of the top thirty phyla of microbial communities in the hemp rhizosphere with different cultivar and fungicide treatments.

Phylum	A	F	D	C	E	K	H	J	L	I	O	G	Average
	Otto2	Otto2	Otto2	BaOX	BaOX	BaOX	Cherry Citrus	Cherry Citrus	Cherry Citrus	Wife	Wife	Wife	
	Fungal inocul.	Natural infect.	Fungicide-treated	Fungal inocul.	Natural infect.	Fungicide-treated	Fungal inocul.	Natural infect.	Fungicide-treated	Fungal inocul.	Natural infect.	Fungicide-treated	
Actinomycetota	27.01	24.39	24.50	30.24	27.06	28.79	25.27	26.68	26.18	22.72	29.42	28.16	26.70
Pseudomonadota	17.63	22.15	15.71	21.10	20.64	18.14	20.70	23.09	19.55	19.06	21.52	17.90	19.77
Acidobacteria	10.57	15.09	14.74	9.50	12.76	11.73	14.72	11.08	13.89	14.93	11.41	10.92	12.61
Archaea_unclassified	10.48	8.52	12.27	7.12	8.50	11.47	9.54	8.98	10.19	11.95	8.98	13.84	10.15
Chloroflexi	12.12	8.83	11.38	10.11	8.99	10.29	9.39	10.19	9.48	9.95	9.17	8.35	9.85
Planctomycetes	8.44	6.14	6.92	5.65	7.27	5.94	4.93	5.50	5.26	5.82	4.45	6.65	6.08
Bacillota	2.69	3.71	3.31	5.19	3.30	3.36	2.84	3.32	2.39	3.08	4.16	2.76	3.34
Gemmatimonadetes	3.39	3.24	1.47	3.36	3.08	1.78	3.49	3.81	3.17	3.07	3.69	2.40	3.00
Verrucomicrobia	2.28	2.21	2.16	1.28	2.91	1.57	2.24	1.95	2.25	1.96	1.60	3.08	2.13
Rokubacteria	1.99	1.96	2.87	1.47	1.66	2.12	2.19	1.46	2.52	2.20	1.41	2.19	2.00
Armatimonadetes	1.04	0.96	0.85	0.77	1.04	0.76	0.98	1.05	1.09	1.18	0.88	1.21	0.99
Bacteroidota	0.52	0.82	0.83	1.11	0.77	0.90	1.35	0.97	1.21	1.07	0.84	0.64	0.92
Crenarchaeota	0.42	0.23	1.22	1.12	0.17	1.40	0.59	0.21	0.81	0.96	0.75	0.05	0.66
Nitrospirae	0.25	0.57	0.63	0.39	0.28	0.52	0.38	0.46	0.44	0.43	0.41	0.34	0.42
Latescibacteria	0.29	0.39	0.23	0.11	0.50	0.21	0.50	0.21	0.43	0.39	0.24	0.41	0.32
Cyanobacteria	0.16	0.27	0.17	0.40	0.36	0.35	0.27	0.33	0.39	0.51	0.29	0.22	0.31
unclassified	0.29	0.12	0.22	0.47	0.17	0.22	0.16	0.16	0.19	0.22	0.25	0.25	0.23
Patescibacteria	0.12	0.10	0.08	0.31	0.23	0.06	0.08	0.31	0.11	0.12	0.17	0.22	0.16
Entotheonellaeota	0.06	0.12	0.21	0.10	0.06	0.20	0.14	0.06	0.22	0.16	0.17	0.11	0.13
Fibrobacteres	0.03	0.05	0.02	0.03	0.06	0.03	0.04	0.03	0.04	0.04	0.03	0.04	0.04
Thaumarchaeota	0.11	0.01	0.00	0.00	0.06	0.00	0.03	0.03	0.00	0.01	0.01	0.10	0.03
BRC1	0.02	0.02	0.03	0.07	0.03	0.02	0.03	0.02	0.03	0.03	0.02	0.02	0.03
GAL15	0.01	0.02	0.05	0.01	0.02	0.01	0.03	0.01	0.02	0.02	0.02	0.06	0.02
Dependentiae	0.01	0.01	0.02	0.00	0.02	0.05	0.03	0.00	0.03	0.02	0.01	0.03	0.02
Elusimicrobia	0.01	0.02	0.02	0.01	0.02	0.02	0.02	0.02	0.03	0.02	0.01	0.03	0.02
Deinococcus-Thermus	0.02	0.01	0.00	0.03	0.01	0.01	0.01	0.02	0.02	0.02	0.03	0.00	0.01
Candidatus_ Saccharibacteria	0.00	0.01	0.01	0.03	0.00	0.01	0.01	0.01	0.02	0.02	0.04	0.00	0.01
WS2	0.01	0.01	0.01	0.00	0.01	0.01	0.02	0.00	0.01	0.02	0.00	0.01	0.01
WPS-2	0.00	0.00	0.00	0.00	0.00	0.00	0.00	0.00	0.00	0.00	0.00	0.00	0.00
FCPU426	0.00	0.00	0.00	0.00	0.00	0.00	0.00	0.00	0.00	0.00	0.00	0.00	0.00
Others	0.03	0.02	0.07	0.04	0.03	0.05	0.03	0.03	0.03	0.02	0.02	0.02	0.03

**Table 2:** The shared significantly regulated Clusters of Orthologous Genes (COG) between fungicide treatment and natural infection in four hemp rhizospheres.

		Otto2	BaOx	Wife	Cherry Dwarf	Up/Down Regulation
Function	Description	logFC	logFC	logFC	logFC	
COG1690	RNA-splicing ligase RtcB, repairs tRNA damage	0.23	0.15	0.17	0.20	Up
COG1085	Galactose-1-phosphate uridylyltransferase	0.23	0.15	0.16	0.15	Up
COG1321	Mn-dependent transcriptional regulator, DtxR family	0.21	0.16	0.12	0.14	Up
COG1331	Uncharacterized conserved protein YyaL, SSP411 family, contains hioredoxin and six-hairpin glycosidase-like domains	0.18	0.14	0.11	0.14	Up
COG0543	NAD(P)H-flavin reductase	0.13	0.12	0.13	0.14	Up
COG0083	Homoserine kinase	0.23	0.14	0.12	0.13	Up
COG1531	Uncharacterized protein, UPF0248 family	−2.57	−1.55	−1.04	−2.36	Down
COG3756	Uncharacterized conserved protein YdaU, DUF1376 family	−1.62	−1.37	−1.30	−1.98	Down
COG5519	Uncharacterized protein, DUF927 family	−1.64	−0.65	−0.80	−1.55	Down
COG1783	Phage terminase large subunit	−0.70	−0.62	−1.88	−1.50	Down
COG3900	Predicted periplasmic protein	−0.88	−0.86	−0.67	−1.21	Down
COG3524	Capsule polysaccharide export protein KpsE/RkpR	−1.06	−0.74	−0.55	−1.15	Down
COG3338	Carbonic anhydrase	−1.18	−0.92	−0.45	−1.12	Down
COG4378	Uncharacterized protein	−1.30	−0.99	−1.14	−0.97	Down
COG4452	Inner membrane protein involved in colicin E2 resistance	−1.11	−0.73	−0.62	−0.87	Down
COG4228	Mu-like prophage DNA circulation protein	−1.24	−1.24	−1.02	−0.78	Down
COG3724	Succinylarginine dihydrolase	−1.19	−0.60	−0.73	−0.78	Down
COG5339	Uncharacterized conserved protein YdgA, DUF945 family	−1.42	−1.16	−0.89	−0.77	Down
COG4384	Mu-like prophage protein gp45	−1.50	−1.28	−1.17	−0.76	Down
COG5316	Uncharacterized protein	−1.08	−0.67	−0.61	−0.75	Down
COG3784	Uncharacterized conserved protein YdbL, DUF1318 family	−1.04	−0.52	−0.68	−0.74	Down
COG3778	Uncharacterized protein YmfQ in lambdoid prophage, DUF2313 family	−1.10	−1.15	−1.04	−0.74	Down
COG4381	Mu-like prophage protein gp46	−1.10	−1.15	−1.04	−0.74	Down
COG5005	Mu-like prophage protein gpG	−1.40	−1.22	−1.04	−0.73	Down
COG4379	Mu-like prophage tail protein gpP	−0.99	−1.07	−1.07	−0.71	Down
COG4386	Mu-like prophage tail sheath protein gpL	−0.99	−1.07	−1.07	−0.71	Down
COG5003	Mu-like prophage protein gp37	−1.88	−1.23	−1.10	−0.71	Down
COG5455	Periplasmic regulator RcnB of Ni and Co efflux	−0.78	−0.45	−0.59	−0.70	Down
COG3299	Uncharacterized phage protein gp47/JayE	−0.68	−0.65	−0.75	−0.70	Down
COG2866	Murein tripeptide amidase MpaA	−0.97	−0.67	−0.46	−0.67	Down
COG3798	Uncharacterized protein	−0.80	−0.57	−0.42	−0.64	Down
COG3819	Uncharacterized membrane protein	−1.24	−0.92	−0.52	−0.64	Down
COG3817	Uncharacterized membrane protein	−1.25	−0.92	−0.51	−0.63	Down
COG3868	Uncharacterized protein	−0.90	−0.43	−0.44	−0.63	Down
COG3577	Predicted aspartyl protease	−0.55	−0.38	−0.44	−0.61	Down
COG5622	Protein required for attachment to host cells	−0.57	−0.33	−0.38	−0.60	Down
COG3853	Uncharacterized conserved protein YaaN involved in tellurite resistance	−0.36	−0.31	−0.51	−0.60	Down
COG5478	Low-affinity Fe/Cu permease	−0.55	−0.34	−0.43	−0.59	Down
COG0027	Formate-dependent phosphoribosylglycinamide formyltransferase (GAR transformylase)	−0.63	−0.39	−0.39	−0.57	Down
COG4525	ABC-type taurine transport system, ATPase component	−0.47	−0.53	−0.37	−0.55	Down
COG3174	Uncharacterized membrane protein, DUF4010 family	−0.93	−0.65	−0.60	−0.55	Down
COG1755	Uncharacterized protein YpbQ, isoprenylcysteine carboxyl methyltransferase (ICMT) family	−0.37	−0.41	−0.44	−0.55	Down
COG3138	Arginine/ornithine N-succinyltransferase beta subunit	−0.94	−0.50	−0.47	−0.53	Down
COG0418	Dihydroorotase	−0.82	−0.46	−0.43	−0.52	Down
COG4960	Flp pilus assembly protein, protease CpaA	−0.66	−0.36	−0.32	−0.50	Down
COG5454	Predicted secreted protein	−0.58	−0.32	−0.41	−0.49	Down
COG2941	Demethoxyubiquinone hydroxylase, CLK1/Coq7/Cat5 family	−0.90	−0.48	−0.38	−0.49	Down
COG3762	Uncharacterized membrane protein	−0.58	−0.26	−0.44	−0.49	Down
COG3159	Uncharacterized conserved protein YigA, DUF484 family	−0.81	−0.39	−0.40	−0.47	Down
COG5317	Uncharacterized protein	−0.46	−0.30	−0.26	−0.46	Down
COG3908	Uncharacterized protein	−0.45	−0.29	−0.29	−0.45	Down
COG3909	Cytochrome c556	−0.53	−0.32	−0.41	−0.44	Down
COG5509	Uncharacterized small protein, DUF1192 family	−0.42	−0.25	−0.32	−0.44	Down
COG3094	Uncharacterized membrane protein SirB2	−0.75	−0.42	−0.50	−0.44	Down
COG4783	Putative Zn-dependent protease contains TPR repeats	−0.69	−0.39	−0.30	−0.44	Down
COG4784	Putative Zn-dependent protease	−0.71	−0.51	−0.38	−0.43	Down
COG5507	Uncharacterized conserved protein YbaA, DUF1428 family	−0.46	−0.25	−0.27	−0.43	Down
COG1448	Aspartate/tyrosine/aromatic aminotransferase	−0.68	−0.49	−0.43	−0.42	Down
COG4765	Uncharacterized protein	−0.49	−0.29	−0.29	−0.42	Down
COG3807	SH3-like domain	−0.52	−0.30	−0.27	−0.42	Down
COG2824	Uncharacterized Zn-ribbon-containing protein	−0.55	−0.46	−0.47	−0.42	Down
COG5447	Uncharacterized protein	−0.48	−0.27	−0.29	−0.42	Down
COG5458	Uncharacterized protein	−0.48	−0.27	−0.30	−0.42	Down
COG4544	Uncharacterized conserved protein	−0.43	−0.27	−0.30	−0.41	Down
COG5481	Uncharacterized protein	−0.47	−0.26	−0.25	−0.41	Down
COG5385	Uncharacterized protein	−0.48	−0.26	−0.32	−0.41	Down
COG5360	Uncharacterized conserved protein, heparinase superfamily	−0.45	−0.25	−0.28	−0.41	Down
COG3128	Predicted 2-oxoglutarate- and Fe(II)-dependent dioxygenase YbiX	−0.86	−0.57	−0.29	−0.40	Down
COG1956	GAF domain-containing protein, putative methionine-R-sulfoxide reductase	−0.48	−0.35	−0.55	−0.40	Down
COG5452	Uncharacterized protein	−0.47	−0.25	−0.31	−0.40	Down
COG5416	Uncharacterized integral membrane protein	−0.34	−0.25	−0.20	−0.40	Down
COG2733	Uncharacterized membrane-anchored protein YjiN, DUF445 family	−0.50	−0.36	−0.31	−0.39	Down
COG2032	Cu/Zn superoxide dismutase	−0.29	−0.23	−0.29	−0.39	Down
COG4120	ABC-type uncharacterized transport system, permease component	−0.22	−0.26	−0.41	−0.39	Down
COG2747	Negative regulator of flagellin synthesis (anti-sigma28 factor)	−0.47	−0.30	−0.29	−0.38	Down
COG5265	ABC-type transport system involved in Fe-S cluster assembly, permease, and ATPase components	−0.56	−0.29	−0.25	−0.37	Down
COG5394	Polyhydroxyalkanoate (PHA) synthesis regulator protein, binds DNA and PHA	−0.56	−0.29	−0.24	−0.37	Down
COG3175	Cytochrome c oxidase assembly protein Cox11	−0.56	−0.30	−0.26	−0.37	Down
COG2932	Phage repressor protein C contains Cro/C1-type HTH and peptidase s24 domains	−0.47	−0.30	−0.25	−0.37	Down
COG2821	Membrane-bound lytic murein transglycosylase	−0.58	−0.28	−0.26	−0.35	Down
COG2935	Arginyl-tRNA—protein-N-Asp/Glu arginylyltransferase	−0.49	−0.25	−0.25	−0.34	Down
COG2827	Predicted endonuclease, GIY-YIG superfamily	−0.46	−0.33	−0.18	−0.34	Down
COG3158	K+ transporter	−0.56	−0.34	−0.23	−0.32	Down
COG1986	Non-canonical (house-cleaning) NTP pyrophosphatase, all-alpha NTP-Ppase family	−0.50	−0.60	−0.56	−0.32	Down
COG2385	Peptidoglycan hydrolase (amidase) enhancer domain	−0.48	−0.55	−0.51	−0.31	Down
COG3169	Uncharacterized conserved protein, DUF486 family	−0.58	−0.43	−0.31	−0.31	Down
COG4145	Na+/panthothenate symporter	−0.53	−0.75	−0.58	−0.31	Down
COG0397	Uncharacterized conserved protein YdiU, UPF0061 family	−0.47	−0.28	−0.25	−0.30	Down
COG2840	DNA-nicking endonuclease, Smr domain	−0.51	−0.23	−0.24	−0.30	Down
COG3920	Two-component sensor histidine kinase, HisKA, and HATPase domains	−0.29	−0.19	−0.15	−0.27	Down
COG0610	Type I site-specific restriction-modification system, R (restriction) subunit, and related helicases	−0.59	−0.46	−0.22	−0.26	Down
COG1281	Redox-regulated molecular chaperone, HSP33 family	−0.38	−0.17	−0.26	−0.23	Down
COG1565	SAM-dependent methyltransferase, MidA family	−0.30	−0.19	−0.15	−0.23	Down
COG0807	GTP cyclohydrolase II	−0.48	−0.32	−0.22	−0.22	Down
COG073′	2′,3’-cyclic-nucleotide 2’-phosphodiesteras’/5’- o’ 3’-nucleotidase 5’-nucleotidase family	−0.29	−0.19	−0.22	−0.19	Down
COG1764	Organic hydroperoxide reductase OsmC/OhrA	−0.19	−0.16	−0.11	−0.13	Down
COG1393	Arsenate reductase and related proteins, glutaredoxin family	−0.23	−0.13	−0.15	−0.11	Down
COG1301	Na+/H+-dicarboxylate symporter	−0.23	−0.19	−0.15	−0.10	Down

Note: All genes differ significantly between fungicide-treated and natural infection estimated by R statical with DEseq2 (Adj. *P* < 0.05). Log(FC): Log of fold change between fungicide treatment and natural infection.

**Table 3:** The shared significantly regulated enzymes between fungicide treatment and natural infection in four hemp rhizospheres.

		Otto II	BaOx	Wife	Cherry Dwarf	Up/Down
Function	Description	logFC	logFC	logFC	logFC	
EC:6.5.1.3	RNA ligase (ATP)	0.22	0.14	0.14	0.18	Up
EC:2.5.1.29	Geranylgeranyl diphosphate synthase	0.17	0.10	0.12	0.12	Up
EC:5.4.2.12	Phosphoglycerate mutase (2,3-diphosphoglycerate-independent)	0.12	0.08	0.05	0.07	Up
EC:1.1.1.276	Serine 3-dehydrogenase (NADP(^+^))	−1.54	−0.78	−0.94	−1.02	Down
EC:1.2.1.71	Succinylglutamate-semialdehyde dehydrogenase	−1.28	−0.74	−0.72	−0.82	Down
EC:1.2.1.24	Succinate-semialdehyde dehydrogenase (NAD(^+^))	−1.88	−1.24	−1.10	−0.80	Down
EC:3.5.3.23	N-succinylarginine dihydrolase	−1.20	−0.61	−0.74	−0.78	Down
EC:1.4.99.6	D-arginine dehydrogenase	−1.10	−0.62	−0.65	−0.75	Down
EC:3.2.1.99	Arabinan endo-1,5-alpha-L-arabinosidase	−0.75	−0.54	−0.57	−0.74	Down
EC:1.3.3.5	Bilirubin oxidase	−1.33	−0.92	−1.01	−0.74	Down
EC:1.1.1.48	D-galactose 1-dehydrogenase	−0.59	−0.41	−0.56	−0.64	Down
EC:1.14.11.2	Procollagen-proline dioxygenase	−0.58	−0.40	−0.63	−0.59	Down
EC:5.2.1.2	Maleylacetoacetate isomerase	−0.72	−0.42	−0.52	−0.55	Down
EC:2.3.1.109	Arginine N-succinyltransferase	−0.95	−0.51	−0.48	−0.54	Down
EC:5.1.1.13	Aspartate racemase	−0.63	−0.32	−0.63	−0.53	Down
EC:2.6.1.57	Aromatic-amino-acid transaminase	−0.98	−0.69	−0.45	−0.50	Down
EC:3.1.2.12	S-formylglutathione hydrolase	−0.71	−0.42	−0.26	−0.47	Down
EC:1.2.1.91	3-oxo-5,6-dehydrosuberyl-CoA semialdehyde dehydrogenase	−0.72	−0.54	−0.32	−0.39	Down
EC:3.3.2.12	Oxepin-CoA hydrolase	−0.72	−0.54	−0.32	−0.39	Down
EC:2.3.2.8	Arginyltransferase	−0.50	−0.26	−0.26	−0.35	Down
EC:6.4.1.4	Methylcrotonoyl-CoA carboxylase	−0.40	−0.23	−0.25	−0.33	Down
EC:3.5.2.9	5-oxoprolinase (ATP-hydrolyzing)	−0.42	−0.22	−0.29	−0.33	Down
EC:2.3.1.223	3-oxo-5,6-didehydrosuberyl-CoA thiolase	−0.73	−0.96	−0.62	−0.33	Down
EC:3.4.11.19	D-stereospecific aminopeptidase	−0.41	−0.36	−0.33	−0.30	Down
EC:5.4.99.28	tRNA pseudouridine(32) synthase	−0.51	−0.36	−0.25	−0.29	Down
EC:5.4.99.29	23S rRNA pseudouridine(746) synthase	−0.51	−0.36	−0.25	−0.29	Down
EC:1.3.3.3	Coproporphyrinogen oxidase	−0.55	−0.29	−0.31	−0.26	Down
EC:3.4.11.5	Prolyl aminopeptidase	−0.40	−0.26	−0.20	−0.21	Down
EC:2.3.1.117	2,3,4,5-tetrahydropyridine-2,6-dicarboxylate N-succinyltransferase	−0.33	−0.17	−0.15	−0.18	Down
EC:3.5.1.28	N-acetylmuramoyl-L-alanine amidase	−0.13	−0.12	−0.17	−0.12	Down
EC:2.7.1.69	Protein-N(pi)-phosphohistidine--sugar phosphotransferase	−0.21	−0.22	−0.09	−0.11	Down
EC:3.1.11.2	Exodeoxyribonuclease III	−0.20	−0.14	−0.09	−0.10	Down

Note: All genes are significantly different between fungicide-treated and natural infection estimated by R statical with DEseq2 (Adj. *P* < 0.05). Log (FC): Log of fold change between fungicide treatment and natural infection.

**Table 4:** The shared significantly regulated pathways between fungicide treatment and natural infection in four hemp rhizospheres.

		Otto II	BaOx	Wife	Cherry Dwarf	Up/Down
Pathways	Description	logFC	logFC	logFC	logFC	
TYRFUMCAT-PWY	L-tyrosine degradation I	−0.77	−0.52	−0.34	−0.37	Down

Note: All genes are significantly different between fungicide-treated and natural infection estimated by R statical with DEseq2 (Adj. *P* < 0.05). Log(FC): Log of fold change between fungicide treatment and natural infection.

## Data Availability

The datasets presented in this study are publicly available at https://submit.ncbi.nlm.nih.gov/subs/genbank/SUB13018760/overview.

## Supplementary Materials

The following supporting information can be downloaded at: https://www.mdpi.com/article/10.3390/ijms25115892/s1.
